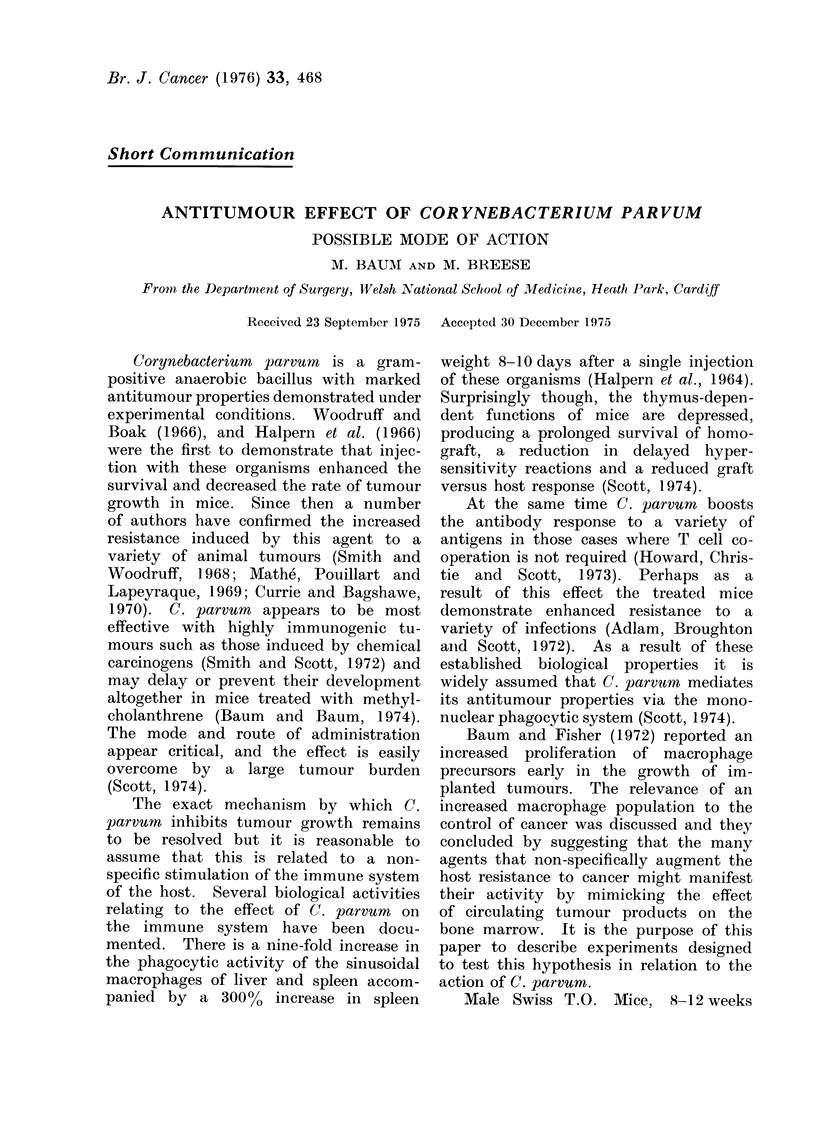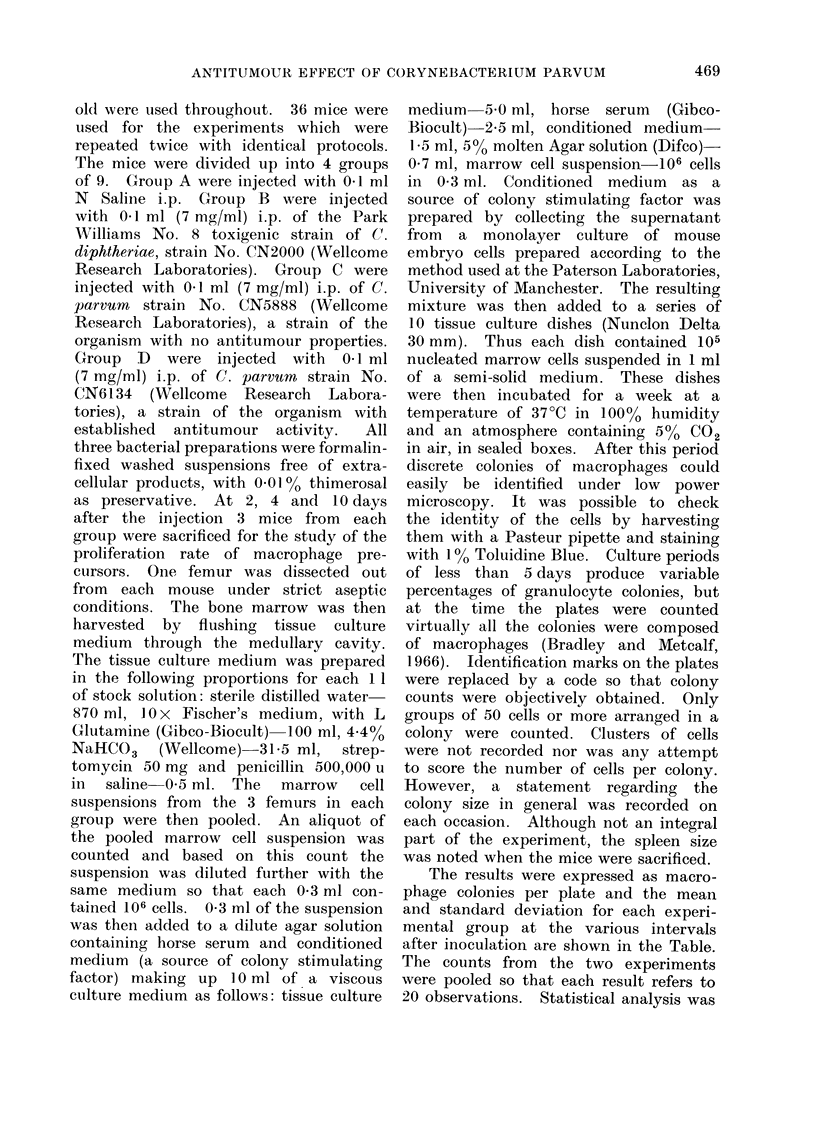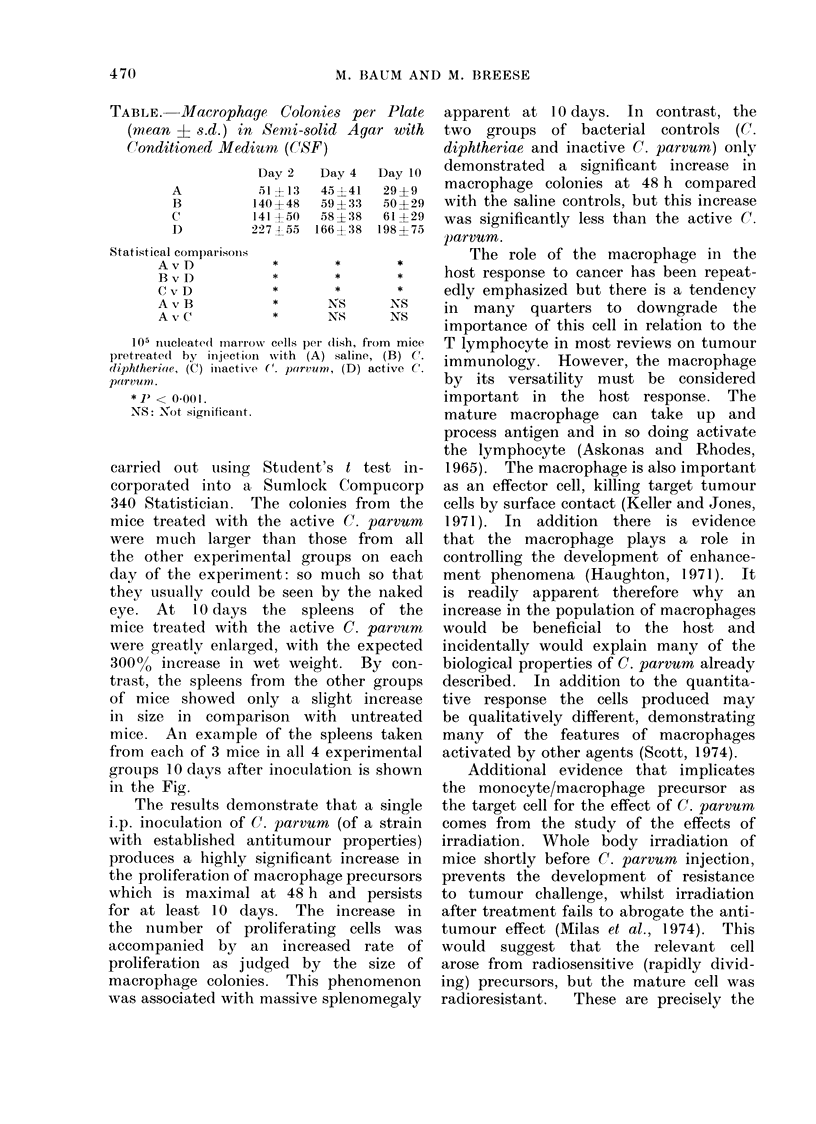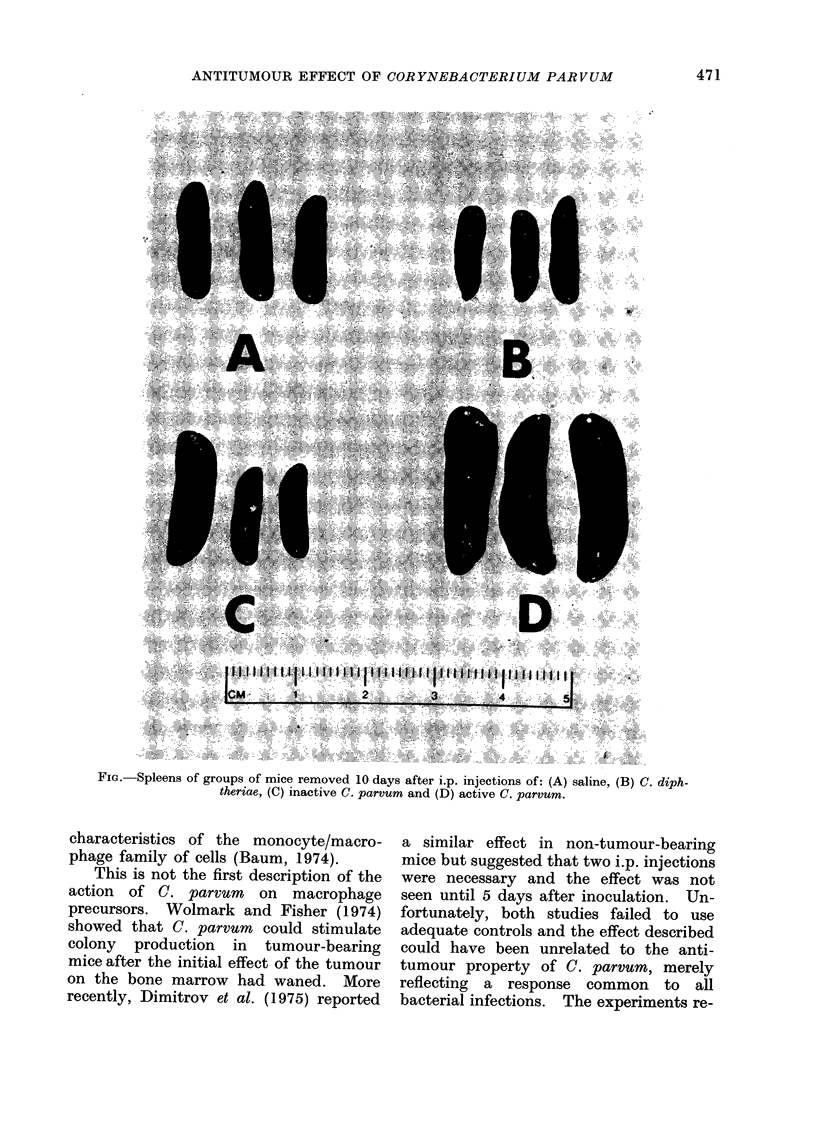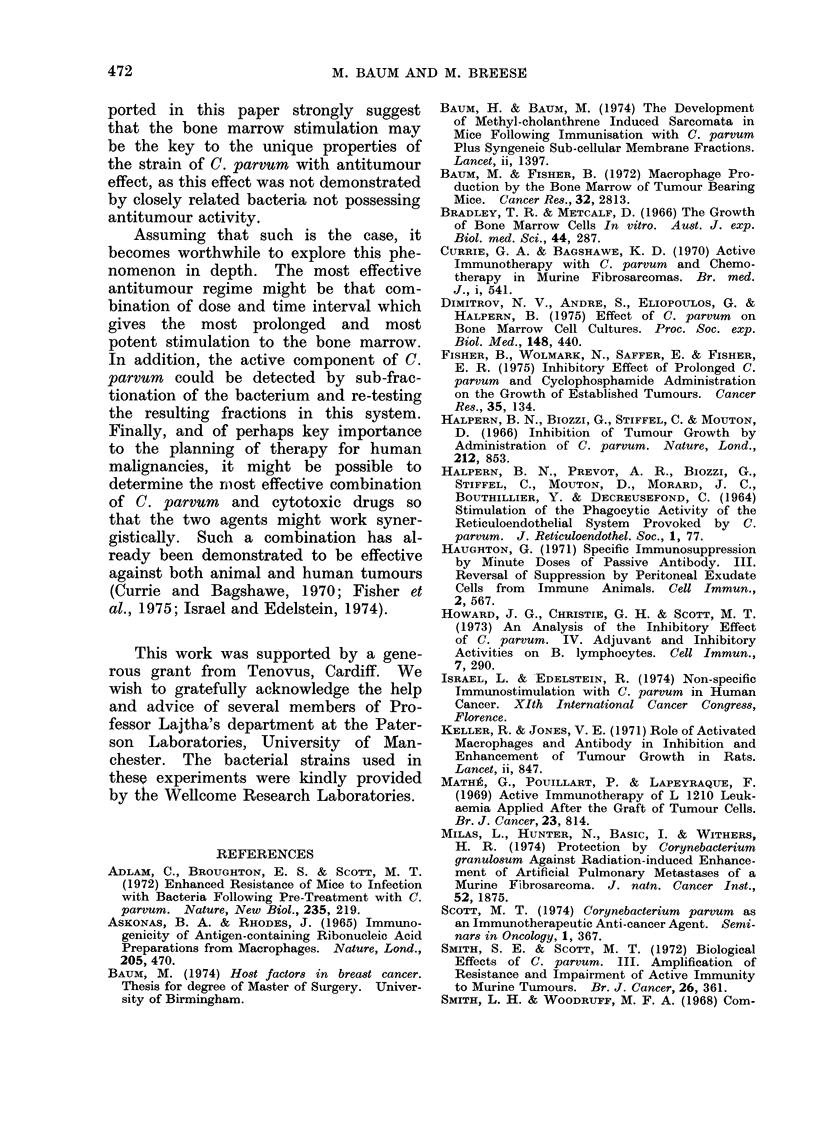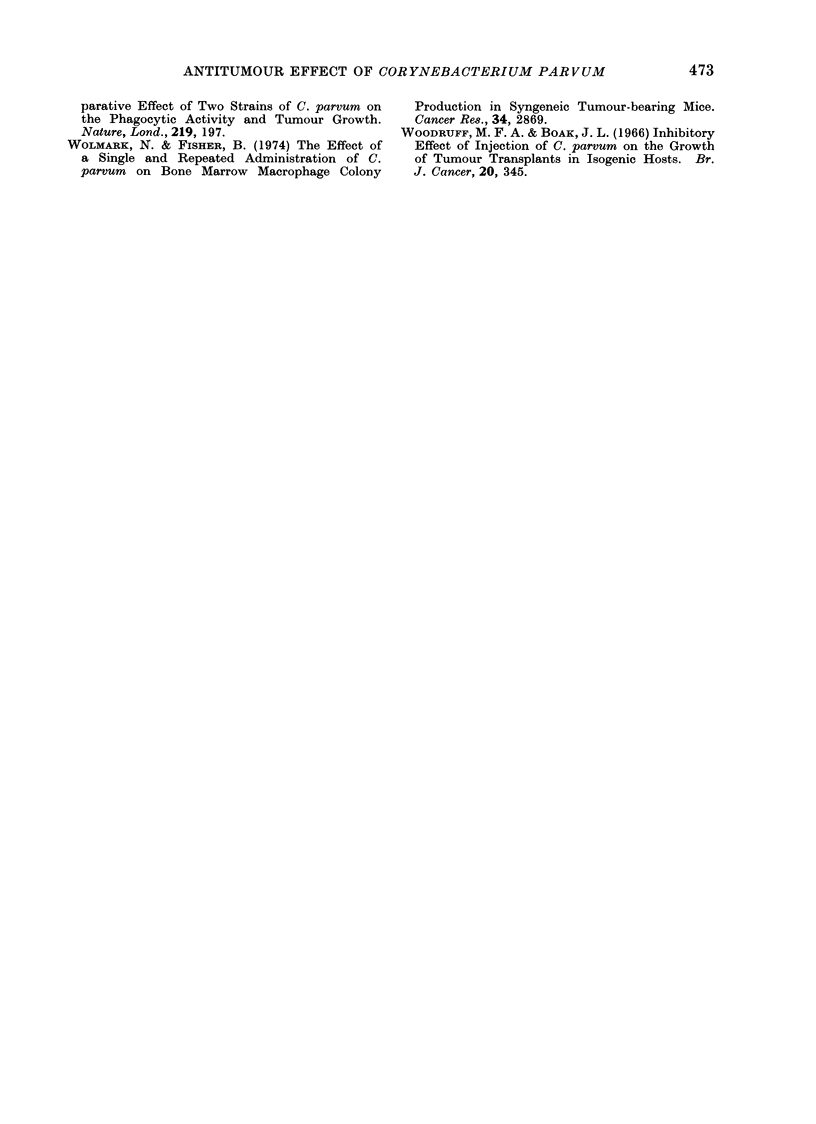# Antitumour effect of corynebacterium parvum. Possible mode of action.

**DOI:** 10.1038/bjc.1976.73

**Published:** 1976-04

**Authors:** M. Baum, M. Breese

## Abstract

**Images:**


Br. J. Cancer (1976) 33, 468

### Short Communication

### ANTITUMOUR EFFECT OF *CORYNEBACTERIUM PARVUM*

### POSSIBLE MODE OF ACTION

M. BAUM AND M. BREESE

*From the Department of Surgery, Welsh National School of Medicine, Heath Park, Cardiff*

Received 23 September 1975 Accepted 30 December 1975

*Corynebacterium parvum* is a gram-positive anaerobic bacillus with marked antitumour properties demonstrated under experimental conditions. Woodruff and Boak (1966), and Halpern *et al.* (1966) were the first to demonstrate that injection with these organisms enhanced the survival and decreased the rate of tumour growth in mice. Since then a number of authors have confirmed the increased resistance induced by this agent to a variety of animal tumours (Smith and Woodruff, 1968; Mathé, Pouillart and Lapeyraque, 1969; Currie and Bagshawe, 1970). *C. parvum* appears to be most effective with highly immunogenic tumours such as those induced by chemical carcinogens (Smith and Scott, 1972) and may delay or prevent their development altogether in mice treated with methylcholanthrene (Baum and Baum, 1974). The mode and route of administration appear critical, and the effect is easily overcome by a large tumour burden (Scott, 1974).

The exact mechanism by which *C. parvum* inhibits tumour growth remains to be resolved but it is reasonable to assume that this is related to a non-specific stimulation of the immune system of the host. Several biological activities relating to the effect of *C. parvum* on the immune system have been documented. There is a nine-fold increase in the phagocytic activity of the sinusoidal macrophages of liver and spleen accompanied by a 300% increase in spleen weight 8–10 days after a single injection of these organisms (Halpern *et al.*, 1964). Surprisingly though, the thymus-dependent functions of mice are depressed, producing a prolonged survival of homograft, a reduction in delayed hypersensitivity reactions and a reduced graft versus host response (Scott, 1974).

At the same time *C. parvum* boosts the antibody response to a variety of antigens in those cases where T cell co-operation is not required (Howard, Christie and Scott, 1973). Perhaps as a result of this effect the treated mice demonstrate enhanced resistance to a variety of infections (Adlam, Broughton and Scott, 1972). As a result of these established biological properties it is widely assumed that *C. parvum* mediates its antitumour properties via the mononuclear phagocytic system (Scott, 1974).

Baum and Fisher (1972) reported an increased proliferation of macrophage precursors early in the growth of implanted tumours. The relevance of an increased macrophage population to the control of cancer was discussed and they concluded by suggesting that the many agents that non-specifically augment the host resistance to cancer might manifest their activity by mimicking the effect of circulating tumour products on the bone marrow. It is the purpose of this paper to describe experiments designed to test this hypothesis in relation to the action of *C. parvum*.

Male Swiss T.O. Mice, 8–12 weeks old were used throughout. 36 mice were used for the experiments which were repeated twice with identical protocols. The mice were divided up into 4 groups of 9. Group A were injected with 0·1 ml N Saline i.p. Group B were injected with 0·1 ml (7 mg/ml) i.p. of the Park Williams No. 8 toxigenic strain of *C. diphtheriae*, strain No. CN2000 (Wellcome Research Laboratories). Group C were injected with 0·1 ml (7 mg/ml) i.p. of *C. parvum* strain No. CN5888 (Wellcome Research Laboratories), a strain of the organism with no antitumour properties. Group D were injected with 0·1 ml (7 mg/ml) i.p. of *C. parvum* strain No. CN6134 (Wellcome Research Laboratories), a strain of the organism with established antitumour activity. All three bacterial preparations were formalin-fixed washed suspensions free of extracellular products, with 0·01% thimerosal as preservative. At 2, 4 and 10 days after the injection 3 mice from each group were sacrificed for the study of the proliferation rate of macrophage precursors. One femur was dissected out from each mouse under strict aseptic conditions. The bone marrow was then harvested by flushing tissue culture medium through the medullary cavity. The tissue culture medium was prepared in the following proportions for each 1 l of stock solution: sterile distilled water—870 ml, 10× Fischer's medium, with L Glutamine (Gibco-Biocult)—100 ml, 4·4% $NaHCO_3$ (Wellcome)—31·5 ml, streptomycin 50 mg and penicillin 500,000 u in saline—0·5 ml. The marrow cell suspensions from the 3 femurs in each group were then pooled. An aliquot of the pooled marrow cell suspension was counted and based on this count the suspension was diluted further with the same medium so that each 0·3 ml contained $10^6$ cells. 0·3 ml of the suspension was then added to a dilute agar solution containing horse serum and conditioned medium (a source of colony stimulating factor) making up 10 ml of a viscous culture medium as follows: tissue culture medium—5·0 ml, horse serum (Gibco-Biocult)—2·5 ml, conditioned medium—1·5 ml, 5% molten Agar solution (Difco)—0·7 ml, marrow cell suspension—$10^6$ cells in 0·3 ml. Conditioned medium as a source of colony stimulating factor was prepared by collecting the supernatant from a monolayer culture of mouse embryo cells prepared according to the method used at the Paterson Laboratories, University of Manchester. The resulting mixture was then added to a series of 10 tissue culture dishes (Nunclon Delta 30 mm). Thus each dish contained $10^5$ nucleated marrow cells suspended in 1 ml of a semi-solid medium. These dishes were then incubated for a week at a temperature of 37°C in 100% humidity and an atmosphere containing 5% $CO_2$ in air, in sealed boxes. After this period discrete colonies of macrophages could easily be identified under low power microscopy. It was possible to check the identity of the cells by harvesting them with a Pasteur pipette and staining with 1% Toluidine Blue. Culture periods of less than 5 days produce variable percentages of granulocyte colonies, but at the time the plates were counted virtually all the colonies were composed of macrophages (Bradley and Metcalf, 1966). Identification marks on the plates were replaced by a code so that colony counts were objectively obtained. Only groups of 50 cells or more arranged in a colony were counted. Clusters of cells were not recorded nor was any attempt to score the number of cells per colony. However, a statement regarding the colony size in general was recorded on each occasion. Although not an integral part of the experiment, the spleen size was noted when the mice were sacrificed.

The results were expressed as macrophage colonies per plate and the mean and standard deviation for each experimental group at the various intervals after inoculation are shown in the Table. The counts from the two experiments were pooled so that each result refers to 20 observations. Statistical analysis was

TABLE.—*Macrophage Colonies per Plate (mean ± s.d.) in Semi-solid Agar with Conditioned Medium (CSF)*

| | Day 2 | Day 4 | Day 10 |
|---|---|---|---|
| A | 51 ± 13 | 45 ± 41 | 29 ± 9 |
| B | 140 ± 48 | 59 ± 33 | 50 ± 29 |
| C | 141 ± 50 | 58 ± 38 | 61 ± 29 |
| D | 227 ± 55 | 166 ± 38 | 198 ± 75 |
| Statistical comparisons | | | |
| A v D | * | * | * |
| B v D | * | * | * |
| C v D | * | * | * |
| A v B | * | NS | NS |
| A v C | * | NS | NS |

$10^5$ nucleated marrow cells per dish, from mice pretreated by injection with (A) saline, (B) *C. diphtheriae*, (C) inactive *C. parvum*, (D) active *C. parvum*.

* $P < 0{\cdot}001$.

NS: Not significant.

carried out using Student's *t* test incorporated into a Sumlock Compucorp 340 Statistician. The colonies from the mice treated with the active *C. parvum* were much larger than those from all the other experimental groups on each day of the experiment: so much so that they usually could be seen by the naked eye. At 10 days the spleens of the mice treated with the active *C. parvum* were greatly enlarged, with the expected 300% increase in wet weight. By contrast, the spleens from the other groups of mice showed only a slight increase in size in comparison with untreated mice. An example of the spleens taken from each of 3 mice in all 4 experimental groups 10 days after inoculation is shown in the Fig.

The results demonstrate that a single i.p. inoculation of *C. parvum* (of a strain with established antitumour properties) produces a highly significant increase in the proliferation of macrophage precursors which is maximal at 48 h and persists for at least 10 days. The increase in the number of proliferating cells was accompanied by an increased rate of proliferation as judged by the size of macrophage colonies. This phenomenon was associated with massive splenomegaly apparent at 10 days. In contrast, the two groups of bacterial controls (*C. diphtheriae* and inactive *C. parvum*) only demonstrated a significant increase in macrophage colonies at 48 h compared with the saline controls, but this increase was significantly less than the active *C. parvum.*

The role of the macrophage in the host response to cancer has been repeatedly emphasized but there is a tendency in many quarters to downgrade the importance of this cell in relation to the T lymphocyte in most reviews on tumour immunology. However, the macrophage by its versatility must be considered important in the host response. The mature macrophage can take up and process antigen and in so doing activate the lymphocyte (Askonas and Rhodes, 1965). The macrophage is also important as an effector cell, killing target tumour cells by surface contact (Keller and Jones, 1971). In addition there is evidence that the macrophage plays a role in controlling the development of enhancement phenomena (Haughton, 1971). It is readily apparent therefore why an increase in the population of macrophages would be beneficial to the host and incidentally would explain many of the biological properties of *C. parvum* already described. In addition to the quantitative response the cells produced may be qualitatively different, demonstrating many of the features of macrophages activated by other agents (Scott, 1974).

Additional evidence that implicates the monocyte/macrophage precursor as the target cell for the effect of *C. parvum* comes from the study of the effects of irradiation. Whole body irradiation of mice shortly before *C. parvum* injection, prevents the development of resistance to tumour challenge, whilst irradiation after treatment fails to abrogate the antitumour effect (Milas *et al.*, 1974). This would suggest that the relevant cell arose from radiosensitive (rapidly dividing) precursors, but the mature cell was radioresistant. These are precisely the

FIG.—Spleens of groups of mice removed 10 days after i.p. injections of: (A) saline, (B) *C. diphtheriae*, (C) inactive *C. parvum* and (D) active *C. parvum*.

characteristics of the monocyte/macrophage family of cells (Baum, 1974).

This is not the first description of the action of *C. parvum* on macrophage precursors. Wolmark and Fisher (1974) showed that *C. parvum* could stimulate colony production in tumour-bearing mice after the initial effect of the tumour on the bone marrow had waned. More recently, Dimitrov *et al.* (1975) reported a similar effect in non-tumour-bearing mice but suggested that two i.p. injections were necessary and the effect was not seen until 5 days after inoculation. Unfortunately, both studies failed to use adequate controls and the effect described could have been unrelated to the antitumour property of *C. parvum*, merely reflecting a response common to all bacterial infections. The experiments reported in this paper strongly suggest that the bone marrow stimulation may be the key to the unique properties of the strain of *C. parvum* with antitumour effect, as this effect was not demonstrated by closely related bacteria not possessing antitumour activity.

Assuming that such is the case, it becomes worthwhile to explore this phenomenon in depth. The most effective antitumour regime might be that combination of dose and time interval which gives the most prolonged and most potent stimulation to the bone marrow. In addition, the active component of *C. parvum* could be detected by sub-fractionation of the bacterium and re-testing the resulting fractions in this system. Finally, and of perhaps key importance to the planning of therapy for human malignancies, it might be possible to determine the most effective combination of *C. parvum* and cytotoxic drugs so that the two agents might work synergistically. Such a combination has already been demonstrated to be effective against both animal and human tumours (Currie and Bagshawe, 1970; Fisher *et al.*, 1975; Israel and Edelstein, 1974).

This work was supported by a generous grant from Tenovus, Cardiff. We wish to gratefully acknowledge the help and advice of several members of Professor Lajtha's department at the Paterson Laboratories, University of Manchester. The bacterial strains used in these experiments were kindly provided by the Wellcome Research Laboratories.

### REFERENCES

ADLAM, C., BROUGHTON, E. S. & SCOTT, M. T. (1972) Enhanced Resistance of Mice to Infection with Bacteria Following Pre-Treatment with *C. parvum*. *Nature, New Biol.*, **235**, 219.

ASKONAS, B. A. & RHODES, J. (1965) Immunogenicity of Antigen-containing Ribonucleic Acid Preparations from Macrophages. *Nature, Lond.*, **205**, 470.

BAUM, M. (1974) *Host factors in breast cancer.* Thesis for degree of Master of Surgery. University of Birmingham.

BAUM, H. & BAUM, M. (1974) The Development of Methyl-cholanthrene Induced Sarcomata in Mice Following Immunisation with *C. parvum* Plus Syngeneic Sub-cellular Membrane Fractions. *Lancet*, ii, 1397.

BAUM, M. & FISHER, B. (1972) Macrophage Production by the Bone Marrow of Tumour Bearing Mice. *Cancer Res.*, **32**, 2813.

BRADLEY, T. R. & METCALF, D. (1966) The Growth of Bone Marrow Cells *In vitro*. *Aust. J. exp. Biol. med. Sci.*, **44**, 287.

CURRIE, G. A. & BAGSHAWE, K. D. (1970) Active Immunotherapy with *C. parvum* and Chemotherapy in Murine Fibrosarcomas. *Br. med. J.*, i, 541.

DIMITROV, N. V., ANDRE, S., ELIOPOULOS, G. & HALPERN, B. (1975) Effect of *C. parvum* on Bone Marrow Cell Cultures. *Proc. Soc. exp. Biol. Med.*, **148**, 440.

FISHER, B., WOLMARK, N., SAFFER, E. & FISHER, E. R. (1975) Inhibitory Effect of Prolonged *C. parvum* and Cyclophosphamide Administration on the Growth of Established Tumours. *Cancer Res.*, **35**, 134.

HALPERN, B. N., BIOZZI, G., STIFFEL, C. & MOUTON, D. (1966) Inhibition of Tumour Growth by Administration of *C. parvum*. *Nature, Lond.*, **212**, 853.

HALPERN, B. N., PREVOT, A. R., BIOZZI, G., STIFFEL, C., MOUTON, D., MORARD, J. C., BOUTHILLIER, Y. & DECREUSEFOND, C. (1964) Stimulation of the Phagocytic Activity of the Reticuloendothelial System Provoked by *C. parvum*. *J. Reticuloendothel. Soc.*, **1**, 77.

HAUGHTON, G. (1971) Specific Immunosuppression by Minute Doses of Passive Antibody. III. Reversal of Suppression by Peritoneal Exudate Cells from Immune Animals. *Cell Immun.*, **2**, 567.

HOWARD, J. G., CHRISTIE, G. H. & SCOTT, M. T. (1973) An Analysis of the Inhibitory Effect of *C. parvum*. IV. Adjuvant and Inhibitory Activities on B. lymphocytes. *Cell Immun.*, **7**, 290.

ISRAEL, L. & EDELSTEIN, R. (1974) Non-specific Immunostimulation with *C. parvum* in Human Cancer. *XIth International Cancer Congress, Florence.*

KELLER, R. & JONES, V. E. (1971) Role of Activated Macrophages and Antibody in Inhibition and Enhancement of Tumour Growth in Rats. *Lancet*, ii, 847.

MATHÉ, G., POUILLART, P. & LAPEYRAQUE, F. (1969) Active Immunotherapy of L 1210 Leukaemia Applied After the Graft of Tumour Cells. *Br. J. Cancer*, **23**, 814.

MILAS, L., HUNTER, N., BASIC, I. & WITHERS, H. R. (1974) Protection by *Corynebacterium granulosum* Against Radiation-induced Enhancement of Artificial Pulmonary Metastases of a Murine Fibrosarcoma. *J. natn. Cancer Inst.*, **52**, 1875.

SCOTT, M. T. (1974) *Corynebacterium parvum* as an Immunotherapeutic Anti-cancer Agent. *Seminars in Oncology*, **1**, 367.

SMITH, S. E. & SCOTT, M. T. (1972) Biological Effects of *C. parvum*. III. Amplification of Resistance and Impairment of Active Immunity to Murine Tumours. *Br. J. Cancer*, **26**, 361.

SMITH, L. H. & WOODRUFF, M. F. A. (1968) Comparative Effect of Two Strains of *C. parvum* on the Phagocytic Activity and Tumour Growth. *Nature, Lond.*, **219**, 197.

WOLMARK, N. & FISHER, B. (1974) The Effect of a Single and Repeated Administration of *C. parvum* on Bone Marrow Macrophage Colony Production in Syngeneic Tumour-bearing Mice. *Cancer Res.*, **34**, 2869.

WOODRUFF, M. F. A. & BOAK, J. L. (1966) Inhibitory Effect of Injection of *C. parvum* on the Growth of Tumour Transplants in Isogenic Hosts. *Br. J. Cancer*, **20**, 345.